# Independent natural genetic variation of punishment- versus relief-memory

**DOI:** 10.1098/rsbl.2016.0657

**Published:** 2016-12

**Authors:** Mirjam Appel, Claus-Jürgen Scholz, Samet Kocabey, Sinead Savage, Christian König, Ayse Yarali

**Affiliations:** 1 Research Group Molecular Systems Biology of Learning, Leibniz Institute for Neurobiology, Magdeburg, Germany; 2 Max Planck Institute of Neurobiology, Martinsried, Germany; 3 Department of Genomics and Immunoregulation, LIMES Institute, University of Bonn, Bonn, Germany; 4 Laboratory for Microarray Applications, IZKF, University of Würzburg, Würzburg, Germany; 5 Center for Behavioral Brain Sciences, Magdeburg, Germany

**Keywords:** associative memory, *Drosophila melanogaster*, natural genetic variation, opponent processes, punishment, relief

## Abstract

A painful event establishes two opponent memories: cues that are associated with pain onset are remembered negatively, whereas cues that coincide with the relief at pain offset acquire positive valence. Such punishment- versus relief-memories are conserved across species, including humans, and the balance between them is critical for adaptive behaviour with respect to pain and trauma. In the fruit fly, *Drosophila melanogaster* as a study case, we found that both punishment- and relief-memories display natural variation across wild-derived inbred strains, but they do not covary, suggesting a considerable level of dissociation in their genetic effectors. This provokes the question whether there may be heritable inter-individual differences in the balance between these opponent memories in man, with potential psycho-clinical implications.

## 1. Background

A painful, traumatic event leaves behind two opponent memories [1]: Cues that come before or during pain later on induce avoidance (in fruit flies: [2–8]), potentiate fear behaviour (in rats and man: [9,10]) and are verbally reported to have negative valence (in man: [9]). Contrarily, cues that coincide with the relief at pain offset are later on approached, they attenuate fear behaviour and, depending on the nature of the task, can be verbally rated as positive [2–11] (for a cross-species review, see [12]). Clearly, a healthy balance between these opponent memories is critical for adaptive behaviour with respect to pain and trauma. Do these memories display natural genetic variation? Do they covary? Can the natural variation, if any, be used to identify candidate genes that distinguish between them or keep them in balance? We turned to the fruit fly as a case to tackle these questions for the first time.

Flies, upon training with an odour that precedes electric shock, learn to avoid this odour as a punishment predictor; whereas an odour that follows shock during training is subsequently approached as it predicts relief [2–7]. We found that both punishment- and relief-memories vary across a comprehensive set of wild-derived inbred fly strains, but do so independently from each other, suggesting significant dissociation in their genetic bases. Indeed, as a first step towards a systematic comparison of punishment- versus relief-memory at the level of the genetic effectors, we ran association analyses between the memory scores and the available transcriptomic/genomic data [13,14] of the inbred strains, yielding candidate genes.

## 2. Results and discussion

**Figure 1.**
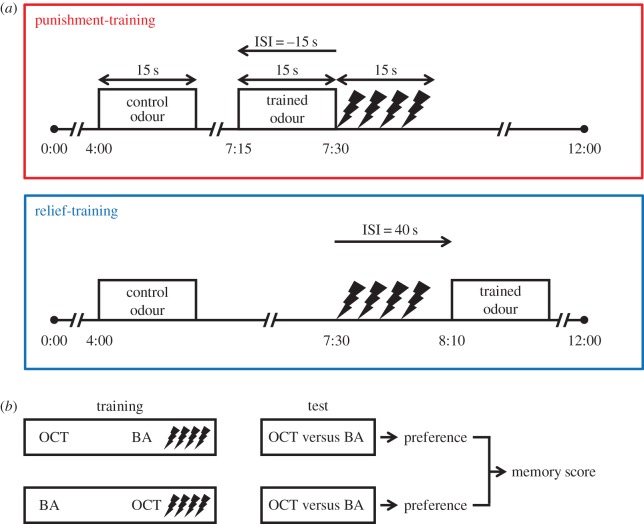
Punishment- versus relief-training. (*a*) One trial each for punishment- and relief-training is depicted. These were identical except for the timing of the trained odour with respect to the electric shock. For punishment-training, the trained odour immediately preceded shock, with an inter-stimulus interval (ISI) of −15 s; whereas for relief-training, the trained odour followed shock with an ISI of 40 s. In both cases, a control odour preceded shock by 3.5 min. (*b*) In each experiment, two subgroups of flies were trained in parallel; for one subgroup 3-octanol was the control odour and benzaldehyde was the trained odour; for the second subgroup the odours were reversed. After training, each subgroup was given the choice between control and trained odours, giving rise to a preference. Based on the preferences of the two subgroups, we calculated a memory score, positive values indicating learned approach, negative ones learned avoidance.

**Figure 2.**
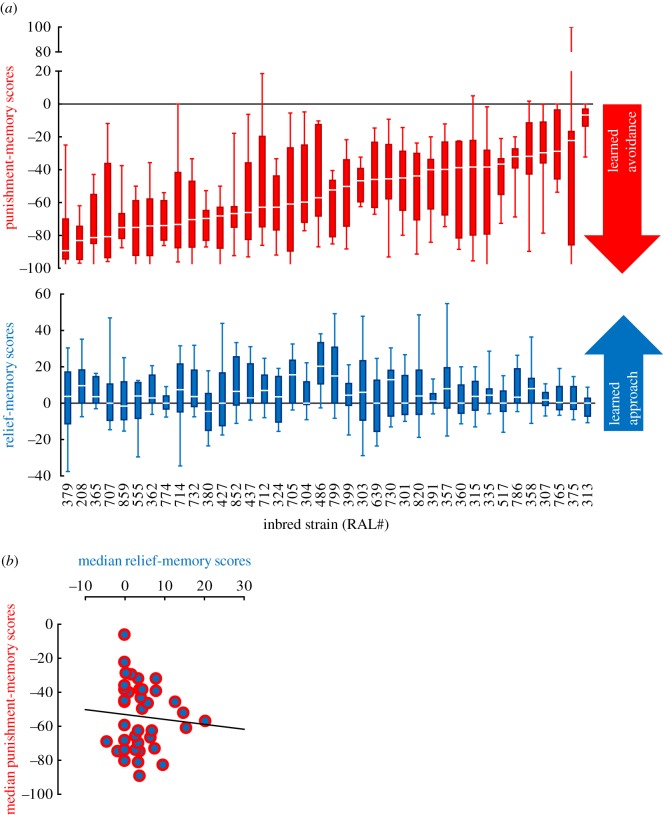
Punishment- and relief-memories varied across inbred strains, but independently from each other. (*a*) Thirty-eight inbred strains differed from each other with respect to both punishment- and relief-memory scores. All strains had significant punishment-memory except RAL#375, which showed a tendency. As for relief-memory, the strains RAL#486, 705, 799, 208, 365, 712 had significant scores, while strains RAL#732, 357, 335, 852, 358, 786, 730, 362, 437 showed tendencies. Box plots show the median as the midline, 25 and 75% as the box boundaries and 10 and 90% as the whiskers. From left to right *N* = 8, 9, 9, 23, 7, 9, 20, 11, 8, 8, 8, 8, 9, 8, 9, 9, 8, 9, 10, 8, 8, 7, 8, 8, 8, 7, 17, 8, 11, 8, 8, 9, 7, 8, 9, 8, 9, 11 for punishment- and 24, 24, 24, 25, 24, 24, 24, 26, 24, 26, 24, 25, 24, 23, 24, 24, 24, 25, 25, 24, 24, 24, 24, 24, 24, 24, 24, 24, 23, 24, 24, 25, 24, 23, 25, 24, 25, 21 for relief-memory. (*b*) Median punishment- versus relief-memory scores did not significantly correlate; *N* = 38.

We characterized 38 wild-derived inbred *Drosophila melanogaster* strains [13,14] in punishment- and relief-memory using paradigms that were parametrically identical, excepting the timing of the trained odour relative to the electric shock (figure 1). Punishment-memory scores were dramatically stronger than relief-memory scores, as was the case in all previous datasets directly comparing these two kinds of memory in the fruit fly [2–8] (see [12] for a meta-analysis). Indeed, among the 38 inbred strains tested, all showed significant punishment-memory, except one strain with a tendency only for punishment-memory; whereas six strains had significant relief-memory, with nine further strains showing a tendency towards it (figure 2*a*, one-sided one-sample Wilcoxon signed-rank tests using a false discovery rate less than 0.05 for significance criterion and *p* < 0.05 for tendency criterion; see electronic supplementary material, table S1 for data and statistical report). This asymmetry in the strength of punishment- versus relief-memory presents a caveat in comparison owing to difference in statistical power. In general, both punishment- and relief-memory scores may have suffered from the extensive inbreeding these strains have undergone [15]. Nevertheless, for either kind of memory we found significant variation across the 38 strains (figure 2*a*, Kruskal–Wallis tests: punishment-memory, *H* = 106.79, d.f. = 37, *p* < 0.0001; relief-memory, *H* = 65.18, d.f. = 37, *p* = 0.0029). Interestingly however, the median scores for either kind of memory did not correlate (figure 2*b*, Pearson correlation: *r*^2^ = 0.0058, *p* = 0.6488).

Thus, punishment- and relief-memories both display natural genetic variation, but they are segregated from each other. This segregation suggests that although particular genes can influence both processes commonly, a considerable portion of the critical genes should be selective for one kind of memory, or exert opposite effects on the two kinds of memory. Indeed, previous reverse genetic analyses had exemplified either kind of scenario: function of *synapsin* is commonly required for punishment- and relief-memory [7]; whereas *white* influences the two kinds of memory oppositely, as its loss-of-function enhances punishment-memory, while suppressing relief-memory [4].

Dissociation of the genetic effectors of punishment- versus relief-memory can, at a very general level, explain the differences between these processes observed so far: In the fruit fly, relief-memories decay more rapidly over time and are less resistant to retrograde amnesia than punishment-memories [6]. Interfering with a particular set of fly dopaminergic neurons using a transgenic blocker of synaptic vesicle recycling impairs punishment-, but not relief-memory [5]. In rats, formation and retrieval of relief-memories require functionality of accumbal reward circuits, rather than amygdalar fear circuits [10,16]. Concordantly, in man, retrieval of punishment- versus relief-associations is accompanied by amygdalar versus striatal activity [10].

Understanding the molecular bases of these differences in punishment- versus relief-memories would be facilitated by a systematic comparison of the respective genetic effectors. To this end, we made use of the natural genetic variation we observed in either kind of memory score. We tested genome-wide for associations between these scores and gene expression levels [13] as well as single nucleotide polymorphisms [14] of the 38 inbred strains. The methodology and results of these analyses are reported in the electronic supplementary material. In brief, we identified 508 and 754 candidate genes for punishment- and relief-memory, respectively, 60 of these being common to both (electronic supplementary material, table S8). Among our candidate genes, those already known for a role in punishment-memory were found (marked in electronic supplementary material, table S8). Clearly, the gene–behaviour relationships we uncovered are only correlative, as is the case in all genome-wide association studies. Owing to the inter-correlations between expression levels of different genes [13] and to the inter-dependency of alleles at different polymorphic loci [14], some candidate genes, although associated with memory, will not be causally related to it. Causal relationships must be independently scrutinized using reverse genetic methods as recently exemplified with respect to a related behaviour, innate escape from electric shock [17]. Such independent validation is especially critical in the present case, given our relatively non-stringent statistical thresholds for candidateship (i.e. no correction for multiple testing was employed). Once verified, however, the candidate genes we identified can indeed provide valuable handles for in depth fly studies on the molecular orchestration of punishment- versus relief-memories, while human orthologues may have roles in pain- and trauma-related behavioural (dys)function.

Natural genetic variation in associative punishment-memory had previously been documented in flies (e.g. [18]) as well as in rodents (e.g. [19]), but never in comparison with relief-memory. Our observation in the fly that these opponent memories *both display heritable variation, without covarying* is novel and thought-provoking: Are there heritable inter-individual differences among man, in terms of a balance between the two kinds of memory? Is such variation, if any, relevant for variation in susceptibility to trauma- and pain-related psychopathologies [1,12]? And if so, what are the critical genetic factors? For this last question, the reverse genetic analyses in the fruit fly to follow up on the present genome-wide approach may provide shortcuts to the answer.

## 3. Material and methods

Memory assays were as previously described [2–7]. Flies were trained and tested *en masse*. Training was repetitive. In each training trial (figure 1*a*), a control odour was presented first; then a pairing of a trained odour and electric shock followed. Punishment- versus relief-training differed only in that the trained odour was, respectively, presented either shortly before shock onset, or shortly after end of shock. Once the training was completed, flies, as a group, were given the choice between the control- and the trained odours. A preference was calculated based on their distribution as
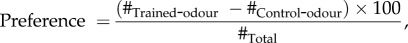
where # indicates the number of flies on each side. Two subgroups of flies were trained and tested in parallel, with reversed roles for 3-octanol and benzaldehyde as the control and trained odour (figure 1*b*). Preferences from the two subgroups were averaged to obtain a memory score as follows:
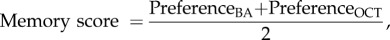
where subscripts of preference indicate the respective trained odour. Positive scores meant learned approach, negative values learned avoidance. We characterized punishment- and relief-memory scores for 38 wild-derived inbred strains from the *Drosophila* Genetic Reference Panel collection [13,14]. For more information, see electronic supplementary material, Methods and results.

## Supplementary Material

Supplementary methods and results

## Supplementary Material

Supplementary tables

## Data accessibility

For data and statistical report, see the electronic supplementary material.
