# Supplementary material for: Independent natural genetic variation of punishment- versus relief-memory
Source: Biol Lett. 2016 Dec;12(12):20160657. doi: 10.1098/rsbl.2016.0657 (PMC5206585; doi:10.1098/rsbl.2016.0657)
Supplement: Supplementary methods and results [file rsbl20160657supp1.docx]

**Independent natural genetic variation of punishment- *versus* relief-memory**

Mirjam Appel^1,2^, Claus-Jürgen Scholz^3,4^, Samet Kocabey^2^, Sinead Savage^2^, Christian König^1^, Ayse Yarali^1,2,5^

^1^ Research Group Molecular Systems Biology of Learning, Leibniz Institute for Neurobiology, Magdeburg, Germany.

^2^ Max Planck Institute of Neurobiology, Martinsried, Germany

^3^ Department of Genomics and Immunoregulation, LIMES Institute, University of Bonn, Bonn, Germany.

^4^ Laboratory for Microarray Applications, IZKF, University of Würzburg, Germany

^5^ Center for Behavioral Brain Sciences, Magdeburg, Germany

Corresponding author: Ayse Yarali, [ayse.yarali@lin-magdeburg.de](mailto:ayse.yarali@lin-magdeburg.de)

**Supplementary methods and results**

**Flies and memory assays**

*Drosophila melanogaster* were kept in mass culture on standard cornmeal-molasses food at 60- 70 % relative humidity and 25 °C temperature under a 12: 12 h light: dark cycle. 1- 3-day-old adults were collected in fresh food bottles and kept under the same culture conditions except at 18 °C temperature at least overnight and at most until they were 4 days old for the experiments. 38 inbred strains from the *Drosophila* Genetic Reference Panel collection were used (available from Bloomington Stock Center). These had been generated by full-sib inbreeding of iso-female strains from Raleigh, North Carolina, USA for more than 20 generations [1, 2].

Experiments took place under homogenous white light, at 23- 25 °C temperature and 60- 80 % humidity. As odourants, 90 μl benzaldehyde (BA) and 340 μl 3-octanol (OCT) (CAS 100-52-7, 589-98-0; both from Fluka, Steinheim, Germany) were applied undiluted into 1 cm-deep Teflon containers of 5 and 14 mm diameters, respectively. Flies were trained and tested in groups of 100- 150. Both punishment- and relief-training used six trials, one of which for each is depicted in Fig. 1A. At time 0:00 min, flies were gently loaded into the experimental setup. From 4:00 min on, the control-odour was presented for 15 s. From 7:30 min on, electric shock was applied as 4 pulses of 100 V; each pulse was 1.2 s- long and was followed by the next with an onset-to-onset interval of 5 s. For punishment-training the trained-odour was presented immediately before electric shock, from 7:15 min on for 15 s, resulting in an inter-stimulus interval (ISI) of -15 s. For relief-training the trained-odour followed electric shock, at 8:10 min (i.e., ISI= 40 s). In either kind of training, at 12:00 min, flies were transferred out of the setup into food vials, where they stayed for 16 min until the next trial. At the end of the sixth training trial, after the usual 16 min break, flies were loaded back into the setup. After a 5 min accommodation period, they were transferred to the choice point between the two odours used during training. After 2 min, the arms of the maze were closed and flies on each side were counted to calculate the preference.

Preference = ( #_Trained-odour_ - #_Control-odour_ ) · 100 / #_Total_

# indicates the number of flies found in the respective maze-arm. Two sub-groups of flies were trained and tested in parallel (Fig. 1B). For one sub-group OCT was the control-odour and BA was trained; for the second sub-group contingencies were reversed. Preferences from the two sub-groups were averaged to obtain a memory score.

Memory score = ( Preference_BA_ + Preference_OCT_ ) / 2

Subscripts of Preference indicate the respective trained-odour. Positive scores reflected learned approach, negative values learned avoidance.

For calculating unisex memory scores, the above equations were applied ignoring the gender of the flies counted. For calculating male or female memory scores, we applied the equations to the flies counted of the respective gender only. In all cases, whenever the total number of flies for calculating a particular Preference was lower than 5, we discarded that Preference value and the respective Memory score. Please note that this led to occasional asymmetry of the sample sizes between male and female memory scores.

Memory scores were analysed using Statistica version 11.0 (StatSoft, Hamburg, Germany) and R version 2.15.1 ([www.r-project.org](http://www.r-project.org)) on a PC. We used non-parametric Kruskal-Wallis tests to probe for differences across the inbred strains, non-parametric one-sided one-sample Wilcoxon signed rank tests to compare scores of each strain to zero, and Pearson correlation to test for relationship between the two kinds of median memory score.

**Association analyses**

We tested genome-wide for associations between on the one hand punishment- as well as relief-memory scores (Fig. 2, Supplemental table 1) and on the other hand gene expression levels [1] as well as single nucleotide polymorphisms [2] of 38 inbred strains. The methodology was as reported in [3] with respect to innate escape from electric shock. We used R version 2.15.1 ([www.r-project.org](http://www.r-project.org)) throughout. For gene expression – memory score association analyses, raw Affymetrix GeneChip Drosophila Genome 2.0 expression microarray data for the 38 inbred strains [1] were downloaded from www.ebi.ac.uk/arrayexpress (accession number EMEXPE-MEXP-1594) using the R Affy package [4]. The raw data covered 18 769 probe-sets and included four expression arrays per strain, two for each gender. For the strain RAL#399 the data from one ‘female’ sample was excluded from analysis, because the distribution of expression levels across the probe-sets rather resembled the typical ‘male’ distribution, deduced from all male samples. For all remaining data, perfect match probe intensity values were pre-processed with variance stabilization normalization (VSN) and summarized with the median polish method to obtain probe-set expression levels using the command ‘vsnrma’ with the default parameter settings [5]. For each probe-set, expression levels were averaged across samples from each strain to obtain mean unisex expression levels. These were then tested for effects on the median unisex punishment- and relief-memory scores (Supplementary table 1) using the following linear model:

Median memory score ~ β0 + β1 · Mean expression level

β0 was the intercept and β1 the estimate for the effect of the mean expression level. β1 was compared to zero with a two-tailed t-test (d.f.= 356). Since none of the probe-sets fulfilled statistical significance using a strict threshold taking into account multiple testing (e.g., *P*< 0.05/ 18 769, corresponding to a Bonferroni correction) for either kind of memory, we considered the 478 and 698 cases with *P*< 0.05 to be suggestive associations to punishment- and relief-memories, respectively. These probe-sets were annotated according to Affymetrix documentation (www.affymetrix.com) and the FlyBase (www.flybase.org) [6] and are listed in Supplementary table 2 for punishment-memory and Supplementary table 5 for relief-memory.

For single nucleotide polymorphism (SNP) – memory score association analyses, the Illumina and 454 SNP calls of the 38 inbred strains [2] were downloaded from http://dgrp.gnets.ncsu.edu/data/. We pre-selected bi-allelic, homo-/ hemizygous SNPs with minor allele frequency (MAF)> 0.1 (calculated over the 38 strains) and call-rate> 0.7. For each such SNP, we tested for an effect on punishment- and relief-memory scores using the following linear model:

Median memory score ~ β0 + β1 · Allele

The minor and major alleles took the values 2 and 0, respectively. β0 was the intercept, whereas β1 was the estimate for the effect of the allele. β1 was subjected to a two-tailed t-test comparing it to zero. With respect to autosomal SNPs, this analysis was done using the unisex memory scores (Supplementary table 1). As no SNP fulfilled the Bonferroni-corrected statistical significance criterion (i.e., *P*< 0.05/ 1 387 514) for either kind of memory, we considered the cases with *P*< 0.0005 to be suggestive associations. With respect to the sex-chromosome SNPs, we did this analysis separately for each sex, using the sex-specific memory scores (Supplementary table 1). Considering the hemizygous state of the males, the male β1 values were multiplied by two. Those SNPs that had *P*< 0.0005 in at least one sex were taken as associated with the respective kind of learning. Altogether, we identified 1096 and 1560 SNPs associated with punishment- and relief-memories, respectively. These were annotated according to *Drosophila melanogaster* reference genome version 5.35 and the FlyBase ([www.flybase.org](http://www.flybase.org)) [6] and are listed in Supplementary tables 3, 4 for punishment-memory and Supplementary tables 6, 7 for relief-memory.

While identifying candidate genes based on gene expression level associations, we excluded the probe-sets with ‘_x_’ or ‘_s_’ qualifiers in their probe-set Affymetrix IDs, as these contain one or more probes that hybridize with products of different genes. Those genes for which at least one corresponding probe-set fulfilled the statistical criterion for association were considered to be candidates. While defining candidate genes based on SNP associations, those SNPs that were annotated to multiple genes were excluded and those genes that had at least one SNP fulfilling the statistical criterion for association were taken as candidates. The suggestive gene expression- and SNP-associations mentioned above respectively pointed to 508 and 754 candidate genes for punishment- and relief-memory, 60 of these genes being common candidates for both (Supplementary table 8). Among our candidate genes, we searched for those with known function in punishment-memory, based on previous studies. Critically, although these studies all investigated punishment-memory, they used various training and testing parameters and did not always exclude non-associative effects in the calculation of the memory scores. In any case, we found 12 “known punishment-memory genes” among our punishment-memory candidates. Interestingly also several “known punishment-memory genes” came up in our study as candidates for relief-memory (Supplementary table 8). In addition, 72 candidate genes from our present approach overlapped with hits from a recent nervous system-wide RNA-interference knock-down screen for effects on punishment-memory (Supplementary table 8).

**Supplementary table legends**

**Supplementary table 1. Punishment- and relief-memory scores**

Punishment- and relief-memory scores for 38 inbred strains are given, based on both gender-specific and unisex calculations. For both punishment- and relief-memory, the median scores were strongly correlated across genders (Pearson correlations: Punishment-memory male *versus* female: r^2^= 0.6692, *P*< 0.0001; Relief-memory male *versus* female: r^2^= 0.2902, *P*= 0.0005). Comparing unisex memory scores to zero revealed significant punishment-memory in all strains but one, which showed a tendency for punishment memory (One-sided one-sample Wilcoxon signed rank tests: FDR< 0.05 as significance criterion and *P*< 0.05 as tendency criterion; all *P* and FDR values given in the table). As for relief-memory, 6 strains showed significantly positive scores; while the scores of 9 further strains had a tendency towards being positive (statistics same as for punishment-memory).

**Supplementary table 2. Gene expression level – punishment-memory associations**

For each probe-set, we tested for a linear regression between the mean expression levels and the median unisex punishment-memory scores. β1 is the respective estimate for the effect of the expression level on punishment-memory. Negative β1 values indicate that the higher the expression level, the stronger the scores; positive β1 values reflect the converse. The t and *P* values refer to the results of a two-tailed t-test comparing β1 to zero. We list probe-sets with *P*< 0.05. Annotations are based on Affymetrix documentation (www.affymetrix.com) and the FlyBase ([www.flybase.org](http://www.flybase.org)) [6].

**Supplementary table 3:** **Autosomal SNP – punishment-memory associations**

For each bi-allelic autosomal SNP with a favourable minor allele frequency and call rate, we tested for a linear regression between the allele type and the median unisex punishment-memory scores. β1 is the respective estimate for the effect of allele type on punishment-memory. The t and *P* values refer to the results of a two-tailed t-test comparing β1 to zero. We list SNPs with *P*< 0.0005. Annotations are based on *Drosophila melanogaster* reference genome version 5.35 and the FlyBase ([www.flybase.org](http://www.flybase.org)) [6].

**Supplementary table 4: X-chromosome-linked SNP – punishment-memory associations**

For each bi-allelic X-chromosome-linked SNP with a favourable minor allele frequency and call rate, we tested for a linear regression between the allele type and either the female or the male median punishment-memory scores. β1, t, and *P* are as explained for Supplementary table 3. We list SNPs with *P*< 0.0005 in at least one gender. Annotations are as explained for Supplementary table 3.

**Supplementary table 5. Gene expression level – relief-memory associations**

All as described in the legend of Supplemental table 2, but for relief-memory. Therefore, positive β1 values indicate that the higher the expression level, the stronger the scores; negative β1 values reflect the converse.

**Supplementary table 6:** **Autosomal SNP – relief-memory associations**

All as described in the legend of Supplemental table 3, but for relief-memory.

**Supplementary table 7: X-chromosome-linked SNP – relief-memory associations**

All as described in the legend of Supplemental table 4, but for relief-memory.

**Supplementary table 8: Candidate genes for punishment- *versus* relief-memory**

Based on the gene expression – memory score as well as SNP – memory score associations listed in Supplemental tables 2- 7, but excluding probe-sets and SNPs with ambiguous annotation, we identified candidate genes for punishment- and/ or relief-memory. We include information as to whether these genes were candidates for innate escape from electric shock [3]; have known role in punishment-memory or were identified as hits in a recent RNA-interference screen for punishment-memory (references in the table).

**Supplementary references**

1. Ayroles JF, Carbone MA, Stone EA, Jordan KW, Lyman RF, Magwire MM, Rollmann SM, Duncan LH, Lawrence F, Anholt RR, et al. 2009 Systems genetics of complex traits in Drosophila melanogaster. Nat Genet. 41, 299–307. (doi:10.1038/ng.332)
2. Mackay TF, Richards S, Stone EA, Barbadilla A, Ayroles JF, Zhu D, Casillas S, Han Y, Magwire MM, Cridland JM, et al. 2012 The Drosophila melanogaster Genetic Reference Panel. Nature 482, 173–178. (doi:10.1038/nature10811)
3. Appel M, Scholz CJ, Müller T, Dittrich M, König C, Bockstaller M, Oguz T, Khalili A, Antwi-Adjei E, Schauer T, et. al. 2015 Genome-Wide Association Analyses Point to Candidate Genes for Electric Shock Avoidance in Drosophila melanogaster. PLoS One. 10, e0126986. (doi:10.1371/journal.pone.0126986)
4. Gautier L, Cope L, Bolstad BM, Irizarry RA. 2004 affy--analysis of Affymetrix GeneChip data at the probe level. Bioinformatics 20, 307-315. (doi:10.1093/bioinformatics/btg405)
5. Huber W, von Heydebreck A, Sultmann H, Poustka A, Vingron M. 2002 Variance stabilization applied to microarray data calibration and to the quantification of differential expression. Bioinformatics 18 Suppl 1, S96-S104.
6. Marygold SJ, Leyland PC, Seal RL, Goodman JL, Thurmond J, FlyBase consortium. 2013 FlyBase: improvements to the bibliography. Nucleic Acids Res 41, D751-757 (doi: 10.1093/nar/gks1024)
7. Tan Y, Yu D, Busto GU, Wilson C, Davis RL. 2013 Wnt signaling is required for long-term memory formation. Cell Rep 4, 1082-1089

(doi: 10.1016/j.celrep.2013.08.007)

1. Huang C, Zheng X, Zhao H, Li M, Wang P, Xie Z, Wang L, Zhong Y. 2012 A permissive role of mushroom body α/β core neurons in long-term memory consolidation in Drosophila. Curr Biol 22, 1981-1989

(doi: 10.1016/j.cub.2012.08.048)

1. Tempel BL, Livingstone MS, Quinn WG. 1984 Mutations in the dopa decarboxylase gene affect learning in Drosophila. Proc Natl Acad Sci U S A 81, 3577-3581
2. Livingstone MS, Sziber PP, Quinn WG. 1984 Loss of calcium/calmodulin responsiveness in adenylate cyclase of rutabaga, a Drosophila learning mutant. Cell 37, 205-215
3. Cowan TM, Siegel RW. 1986 Drosophila mutations that alter ionic conduction disrupt acquisition and retention of a conditioned odor avoidance response. J Neurogenet 3, 187-201.
4. Lee PT, Lin HW, Chang YH, Fu TF, Dubnau J, Hirsh J, Lee T, Chiang AS. 2011 Serotonin-mushroom body circuit modulating the formation of anesthesia-resistant memory in Drosophila. Proc Natl Acad Sci U S A 108, 13794-13799

(doi: 10.1073/pnas.1019483108)

1. Qian M, Pan G, Sun L, Feng C, Xie Z, Tully T, Zhong Y. 2007 Receptor-like tyrosine phosphatase PTP10D is required for long-term memory in Drosophila. J Neurosci 27, 4396-4402.

(doi: 10.1523/JNEUROSCI.4054-06.2007)

1. Ge X, Hannan F, Xie Z, Feng C, Tully T, Zhou H, Xie Z, Zhong Y. 2004 Notch signaling in Drosophila long-term memory formation. Proc Natl Acad Sci U S A 101, 10172-10176

(doi: 10.1073/pnas.0403497101)

1. Chambers DB, Androschuk A, Rosenfelt C, Langer S, Harding M, Bolduc FV. 2015 Insulin signaling is acutely required for long-term memory in Drosophila. Front Neural Circuits 9, 8

(doi: 10.3389/fncir.2015.00008)

1. Didelot G, Molinari F, Tchénio P, Comas D, Milhiet E, Munnich A, Colleaux L, Preat T. 2006 Tequila, a neurotrypsin ortholog, regulates long-term memory formation in Drosophila. Science 313, 851-853

(doi: 10.1126/science.1127215)

1. Blumröder R, Glunz A, Dunkelberger BS, Serway CN, Berger C, Mentzel B, de Belle JS, Raabe T. 2016 Mcm3 replicative helicase mutation impairs neuroblast proliferation and memory in Drosophila. Genes Brain Behav 15, 647-659

(doi: 10.1111/gbb.12304)

1. Li Q, Zhang X, Hu W, Liang X, Zhang F, Wang L, Liu ZJ, Zhong Y. 2016 Importin-7 mediates memory consolidation through regulation of nuclear translocation of training-activated MAPK in Drosophila. Proc Natl Acad Sci U S A 113, 3072-3077 (doi: 10.1073/pnas.1520401113)
2. Wu CL1, Shih MF, Lai JS, Yang HT, Turner GC, Chen L, Chiang AS. 2011 Heterotypic gap junctions between two neurons in the drosophila brain are critical for memory. Curr Biol 21, 848-854

(doi: 10.1016/j.cub.2011.02.041)

1. LaFerriere H, Guarnieri DJ, Sitaraman D, Diegelmann S, Heberlein U, Zars T. 2008 Genetic dissociation of ethanol sensitivity and memory formation in Drosophila melanogaster. Genetics 178, 1895-18902

(doi: 10.1534/genetics.107.084582)

1. Hirano Y, Masuda T, Naganos S, Matsuno M, Ueno K, Miyashita T, Horiuchi J, Saitoe M. 2013 Fasting launches CRTC to facilitate long-term memory formation in Drosophila. Science 339, 443-446

(doi: 10.1126/science.1227170)

1. Akalal DB, Yu D, Davis RL. 2011 The long-term memory trace formed in the Drosophila α/β mushroom body neurons is abolished in long-term memory mutants. J Neurosci 31, 5643-5647

(doi: 10.1523/JNEUROSCI.3190-10.2011)

1. Turrel O, Lampin-Saint-Amaux A, Préat T, Goguel V. 2016 Drosophila Neprilysins Are Involved in Middle-Term and Long-Term Memory. J Neurosci 36, 9535-9546 (doi: 10.1523/JNEUROSCI.3730-15.2016)
2. Volders K, Scholz S, Slabbaert JR, Nagel AC, Verstreken P, Creemers JW, Callaerts P, Schwärzel M. 2012 Drosophila rugose is a functional homolog of mammalian Neurobeachin and affects synaptic architecture, brain morphology, and associative learning. J Neurosci 32, 15193-15204

(doi: 10.1523/JNEUROSCI.6424-11.2012)

1. Tan Y, Yu D, Pletting J, Davis RL. 2010 Gilgamesh is required for rutabaga-independent olfactory learning in Drosophila. Neuron 67, 810-820

(doi: 10.1016/j.neuron.2010.08.020)

1. Walkinshaw E, Gai Y, Farkas C, Richter D, Nicholas E, Keleman K, Davis RL. 2015 Identification of genes that promote or inhibit olfactory memory formation in Drosophila. Genetics 199, 1173-1182

(doi: 10.1534/genetics.114.173575)

1. Bolduc FV, Bell K, Cox H, Broadie KS, Tully T. 2008 Excess protein synthesis in Drosophila fragile X mutants impairs long-term memory. Nat Neurosci 11, 1143-1145

(doi: 10.1038/nn.2175)
